# Performance Assessment of Large Language Models in Medical Consultation: Comparative Study

**DOI:** 10.2196/64318

**Published:** 2025-02-12

**Authors:** Sujeong Seo, Kyuli Kim, Heyoung Yang

**Affiliations:** 1 Future Technology Analysis Center Korea Institute of Science and Technology Information Seoul Republic of Korea; 2 Postal Savings & Insurance Development Institute Seoul Republic of Korea

**Keywords:** artificial intelligence, biomedical, large language model, depression, similarity measurement, text validity

## Abstract

**Background:**

The recent introduction of generative artificial intelligence (AI) as an interactive consultant has sparked interest in evaluating its applicability in medical discussions and consultations, particularly within the domain of depression.

**Objective:**

This study evaluates the capability of large language models (LLMs) in AI to generate responses to depression-related queries.

**Methods:**

Using the PubMedQA and QuoraQA data sets, we compared various LLMs, including BioGPT, PMC-LLaMA, GPT-3.5, and Llama2, and measured the similarity between the generated and original answers.

**Results:**

The latest general LLMs, GPT-3.5 and Llama2, exhibited superior performance, particularly in generating responses to medical inquiries from the PubMedQA data set.

**Conclusions:**

Considering the rapid advancements in LLM development in recent years, it is hypothesized that version upgrades of general LLMs offer greater potential for enhancing their ability to generate “knowledge text” in the biomedical domain compared with fine-tuning for the biomedical field. These findings are expected to contribute significantly to the evolution of AI-based medical counseling systems.

## Introduction

### Overview

The COVID-19 pandemic has brought significant transformations to health care systems worldwide [1]. Additionally, the utilization of artificial intelligence (AI) in the biomedical field has surged [2], and the adoption of natural language processing (NLP) techniques for analyzing or predicting medical data has notably increased [3-5]. Ong et al [6] utilized machine learning techniques on radiographic text to identify the presence, location, and acuity of ischemic strokes. Since the advent of ChatGPT (OpenAI), numerous studies have highlighted the potential impact of generative models across medical domains, including medicine, medical devices, and medical education. Large language models (LLMs) are anticipated to become a cornerstone in the future of health informatics research [7-12].

The COVID-19 pandemic has exacerbated depression, which is widely acknowledged as a significant social and medical concern [13-15]. During the pandemic and the resultant lockdowns, social isolation and withdrawal became prevalent worldwide, leading to the coining of the term “Corona Blue” to describe depression caused by the pandemic, particularly related to self-isolation and social distancing [16]. Even before the pandemic, depression was recognized as a societal issue and a mental health concern with substantial economic implications in many countries [17-22].

Generative AI has recently been employed as an interactive consultant, sparking interest in evaluating its applicability in medical discussions and consultations, particularly in the context of depression. This study aims to assess the suitability of generative AI by comparing the similarity between responses generated by AI models and those provided by humans to depression-related questions. To this end, we collected a set of depression-related questions and corresponding human answers and utilized 4 LLMs—BioGPT [23], PMC-LLaMA [24], ChatGPT [25], and Llama2 [26]—to generate responses.

Questions about depression or depressive disorders can arise from various sources, including validated inquiries recommended by professionals and questions posted online by individuals seeking to understand their symptoms. Furthermore, when selecting an LLM, it is essential to consider 2 key factors. First, is the model domain-specific? This pertains to a fine-tuned pretrained model tailored to the medical domain. Second, is the model a general-purpose intelligent system known for its proficient question-answering capabilities?

Building on this foundation, we designed a basic experiment in which medical questions related to depression were sourced from diverse origins and presented to LLMs for answers. Specifically, we explored the fundamental concepts underlying LLMs and examined the attributes of fine-tuned models within the medical domain. The primary objectives of this study were to evaluate the LLMs’ ability to respond to medical queries, assess the similarity between their answers and those provided by humans, and investigate the differences between domain-specific and general-purpose models.

The main contributions of this study are as follows. First, semantic similarity analysis highlights discrepancies between human expert answers and the knowledge outputs of LLMs. Second, it enables researchers to evaluate the quality of LLM-generated responses by comparing them with human answers. Finally, the experiments show that the latest versions of LLMs outperform earlier iterations, particularly when fine-tuned on specific topics.

### Background and Related Work

#### Large Language Models

The Transformer architecture [27] has significantly influenced the proliferation of LLMs, giving rise to 2 prominent pillars in NLP: GPT and bidirectional encoder representations from transformers (BERT). Qiu et al [25,28] assessed ChatGPT as heralding a new era in the development and deployment of large AI models. Additionally, they observed that the size, generalization, and scale of training/pretraining for general-domain models have increased, thereby enhancing the capacity of a single model. ChatGPT [25], introduced in 2022, offers notable advantages by producing human-like results and being user-friendly and accessible. The development and dissemination of LLMs began in late 2022 with ChatGPT [25], and numerous models followed in 2023 after the release of Llama, which was freely distributed by Meta. Additionally, Meta announced and distributed Llama-2, which is also available for commercial use [26,28,29]. Furthermore, Google introduced its chatbot BARD [30], which was swiftly followed by Alpaca 7B [31], Vicuna [32], and others, all built upon the free Llama model.

#### Health Care Domain Large Language Models

ChatGPT, powered by the GPT-3 model, has successfully navigated all stages of the United States Medical Licensing Examination (USMLE) [11,33]. Concurrently, there has been a rise in LLMs fine-tuned for specific domains, designed to maximize performance in general domains. Specialized models, such as BioBERT and PubMedBERT, which fine-tune existing BERT models using extensive medical domain data, have seen increased development.

LLMs initially introduced in the general domain can undergo additional training with health care domain data (biomedical or biomedicine), resulting in pretrained language models. These pretrained models, tailored to the health care domain, are specialized for tasks such as answering health care–related questions [23,24,34,35] and have evolved to facilitate medical diagnoses through the analysis of medical images.

Furthermore, alongside the development and deployment of LLMs, ongoing research evaluates the performance of both pretrained and general-domain LLMs. For instance, studies have compared the performance of GPT-4, a general-domain LLM, with its predecessor GPT-3.5 and Med-PaLM, a model pretrained specifically in the medical domain [11].

Recent research in the field of medicine, particularly focusing on depression, has explored the application of LLMs such as ChatGPT and Claude, developed by Anthropic. Several publications have introduced methods for evaluating the potential use of LLMs in depression treatment and screening [36-39]. Heston [40] discussed the risks associated with using LLMs in mental health support, particularly for depression. These studies have demonstrated that LLMs can accurately categorize symptoms of depression and anxiety, highlighting their potential for integration into the health care field.

## Methods

### Overview

This section is divided into 3 parts: (1) an overview of the design of experiments; (2) an introduction to medical question-answer data sets and the outputs from our experiments; and (3) a comparison between GPT and Llama models. A detailed process was developed in this study to evaluate the similarity between the answers or outputs generated by LLMs and the original answers. Our methodology consisted of 2 main steps: first, we constructed a data set of depression-related questions and answers sourced from various outlets; second, we segregated the questions to be input into the fine-tuned model from those intended for the general LLMs, based on the data set’s source.

### Ethics Considerations

All data used in this study were from public literature data obtained from PubMed and Quora data. Therefore, ethical approval was not required.

### Experimental Design

**Figure 1:**
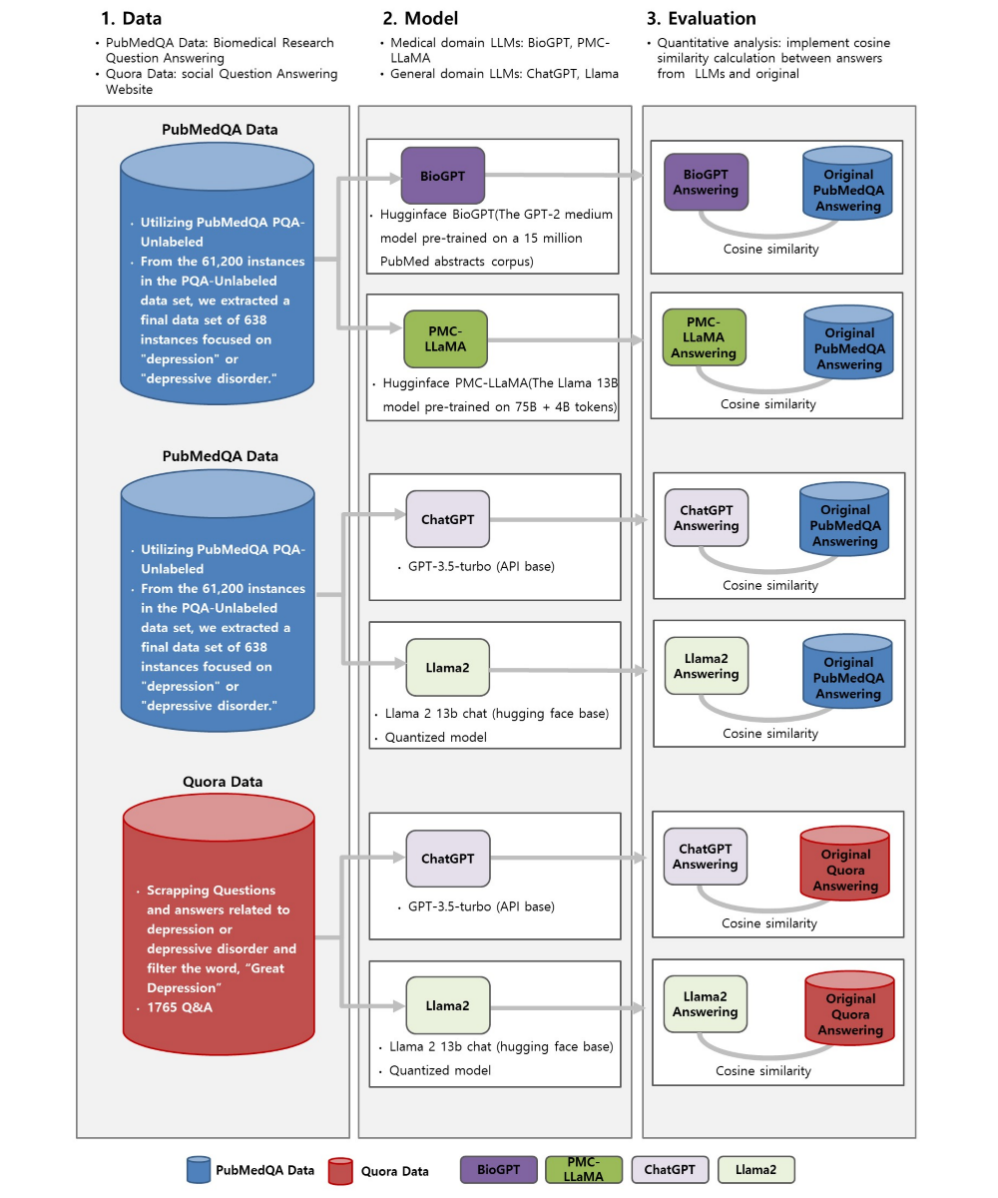
Schematic overview of the data, model, and evaluation in the designed experiment. LLM: large language model.

Figure 1 provides a schematic overview of the study design. This study comprised 3 components: data, model, and evaluation. The model varied based on the type of data, while the evaluation method remained consistent throughout. The data were categorized into 2 types: PubMedQA, derived from medical research abstracts, and question-and-answer data extracted from Quora [41-43], a social platform where users ask and answer questions. The models used in the experiment included 2 types: a basic model that had undergone pretraining and a model fine-tuned with medical data. To evaluate the generated answers from each model, we examined both the quantity and quality of the responses in relation to the input questions. Subsequently, we evaluated the similarity of the generated answers to the correct answers. BERT similarity [36] and SpaCy similarity [37] were used to measure contextual similarities between the human-provided original answers and the LLM-generated responses for each depression-related question.

Various validation metrics can be applied to text generation experiments. He et al [44] introduced common evaluation metrics, including ROUGE, BLEU, METEOR, SACREBLEU, and BERTScore, to assess the quality of LLMs’ responses for electronic health records and laboratory test results. ROUGE, BLEU, and METEOR evaluate similarity in tasks such as full-text comparison, translation, and summarization [38,39,45,46]. By contrast, BERTScore emphasizes semantic similarity rather than relying solely on word matches, such as n-gram overlap. However, this study was designed to quantify similarity within specific contexts and nuances. Therefore, we relied on semantic similarity measures such as BERT and SpaCy as automatic and quantifiable metrics.

Semantic similarity is a relative measure of how similar or dissimilar a new word is to established words. The assumption that semantically related words behave similarly allows for the generalization of user semantic similarity. In the vector space model, similarity is calculated using the cosine measure or normalized correlation coefficient. This is known as vector similarity or cosine similarity [47]. Based on the Euclidean dot product formula, cosine similarity can be defined as follows:



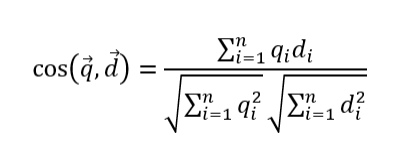



where 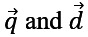 are given n-dimensional vectors of attributes and *i*th components of vectors 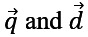, respectively.

Normalized cosine similarity, like other correlation coefficients, is transformed into a value within the range of –1 to 1. A value of –1 indicates that the 2 vectors are diametrically opposed (180° apart), while a value of 0 indicates that the vectors are orthogonal (perpendicular at 90°). Conversely, a value of 1 denotes that the vectors are perfectly aligned in the same direction (horizontal) [47,48].

Furthermore, utilizing automatic context evaluation can replace real human expert feedback. Murty et al [49] suggested that LLM-generated novel personas could be used in data construction. Additionally, Ficler and Goldberg [50] introduced a Delphi expert AI–infused panel, demonstrating its potential to complement human expertise. These studies proposed substituting a real human expert with a persona expert. In this study, a persona expert evaluated the results from LLMs beyond semantic similarity. The persona expert prompt includes the following:

The input Excel (Microsoft Corporation) file contains a question about depression (QUESTION), along with the following columns: expert answer (ANSWER), answer generated by generative AI PMC Llama (PMC_LLAMA_Answer), answer generated by generative AI BIOGPT (biogpt_answer), answer generated by generative AI GPT (GPT_ANSWER), and answer generated by generative AI Llama2 (Llama2_Answer). The question is as follows: “Please compare the answers generated by the generative AI models with the expert answer (ANSWER) using 3 persona agents in the field of mental health, and provide your expert evaluation in an Excel file.” Expert evaluations will be categorized into 3 significance levels: high medical, moderate, and low.

### Data

As summarized in Table 1, PubMedQA contains 638 depression-related questions, while 1763 depression-related questions were extracted from Quora. PubMedQA is a data set of medical questions and answers collected from the text of articles indexed in the PubMed database. If an article’s title poses a question and its abstract is structured with sections such as “Introduction,” “Results,” and “Conclusion,” the abstract can be considered as addressing the question posed in the title. PubMedQA is curated by extracting medical questions and answers from article titles and structured abstracts based on these characteristics. For this study, we extracted 638 and 1764 question-and-answer pairs containing the keywords “depression” or “depressive disorder” from PubMedQA and Quora, respectively. Quora allows community users to freely provide answers, resulting in multiple responses to a single question. We used the “upvotes” feature on Quora to pair each question with the answer that received the highest number of upvotes, selecting the most favored answer as the representative. Both data sets are categorized into 10 major categories and 4 subcategories, as shown in Table 2.

**Table 1:** Summary of the medical question-answers data sets.

Summary	PubMedQA	QuoraQA
Number of questions used in this study, n	638	1763
Source (question-answers)	List of questions and answers containing depression-related keywords in questions from PubMedQA	List of questions and answers containing depression-related keywords in questions from Quora
Prompt (explanations)	Prompt engineering was applied. Question: You are a doctor and I am a patient. Please answer the question within 500 words for length, and as a dialog for format, and professionally. “QUESTION”	None Question: You are a doctor and I am a patient. Please answer the question within 500 words for length, and as a dialog for format, and professionally. “QUESTION”

**Table 2:** Detailed categories of the data sets (same categories applied to both data sets).

Type	Group and subgroup names			Definition
1	Suicide and Risk Factors			Questions about suicide attempts, suicide prevention, and associated risk factors
2	Medications and Treatment Effects			Discussions on the effectiveness of antidepressants, therapeutic interventions, and treatment outcomes
3	Role and Awareness of Health Care Professionals			Insights into how medical professionals diagnose, treat, and improve access to mental health care
4	Inflammation and Immune Response			The relationship between inflammation, immune system activity, and depression
	**Comorbid Disorders**			
5		Anxiety Disorders	Co-occurrence of depression with anxiety or panic disorders	
		Bipolar Disorder	Interrelation between depression and bipolar disorder	
		Physical Illnesses	Links between depression and chronic conditions such as diabetes or cardiovascular diseases	
		Other Mental Disorders	Depression alongside posttraumatic stress disorder, obsessive-compulsive disorder, or schizophrenia	
6	Economic Impact			The financial burden of depression, including treatment costs and workplace productivity
7	Clinical Symptoms			The manifestation, severity, and variations in symptoms of depression
8	Physical Impact			Effects of depression on physical health, such as weight changes and somatic complaints
9	Psychological Factors			The influence of genetic predisposition, family history, and environmental factors on depression
10	Brain and Biological Mechanisms			Studies of structural changes in the brain, neurotransmitter imbalances, and biological pathways related to depression
11	Others			Questions that do not belong to any of the aforementioned categories

### Models

#### Medical Domain LLMs

As outlined in the “Background and Related Work” section, 2 primary types of LLMs were utilized in this study. First, we used medical domain LLMs, which are basic language models (such as Llama or GPT) pretrained for general language tasks and then fine-tuned with a biomedical domain–specific model. Among the various models, BioGPT [23] and PMC-LLaMA [24] were selected for their specialized focus on biomedical text generation and mining.

BioGPT underwent prompt-based fine-tuning using biomedical knowledge and a data set of 15 million PubMed abstracts to perform downstream tasks in NLP, such as relation extraction, question answering, and document classification, based on the GPT-2 model [23].

However, PMC-LLaMA underwent a 2-step training process. First, the Llama 13B model was trained using medical academic papers and medical books for knowledge injection. Subsequently, it underwent instruction tuning for the medical domain. Biomedical papers with PubMed Central IDs were extracted from S2ORC [51], an English-language data set of academic papers, and text content was extracted from PDF versions of books to refine the data set. Samples from RedPajama-Data, a general language corpus, were combined with the aforementioned data sets in a ratio of 15:4:1 (books:papers:general) to create a comprehensive medical domain data set for training a medical-specific knowledge base. Following this, medical-specific instruction tuning was performed, utilizing data from medical conversations, medical rationale question answering, and medical knowledge graph prompting.

Given that BioGPT and PMC-LLaMA are prompt-based models, we employed a prompt engineering format to fully leverage their capabilities. When inputting PubMedQA questions into BioGPT and PMC-LLaMA, the following prompt was appended: “You are a medical doctor. Answer the following question as a medical doctor.”

#### General LLMs

For the comparison group in the biomedical domain, we selected the GPT-3.5-Turbo model and the Llama2 chat model, which were among the most advanced models available from the GPT and Llama series at the time of the experimental design.

GPT-3.5-Turbo (deprecated by OpenAI as of May 2024) is a closed-source, human-like natural language text generation model developed by OpenAI. While the detailed training process is not publicly disclosed, it is known to have a knowledge level sufficient to pass examinations such as the US Bar Examination and the USMLE. The GPT-3.5-Turbo model has been extensively used in ChatGPT and has demonstrated exceptional abilities across various domains, including mathematical reasoning, coding, and human interaction, such as understanding language and engaging in conversation [33,52,53].

Llama2, developed by Meta, consists of a collection of open-source pretrained and fine-tuned LLMs, ranging in size from small to large, with parameters spanning from 7 billion to 70 billion. Unlike the previous Llama model, which was available only for research purposes, Llama2 can be used for commercial applications. Meta has released a version of Llama2-Chat specifically designed for dialogue use cases. Llama2-Chat is available in 7B, 13B, and 70B parameter configurations, with the 13B model used in this study. According to a study by Touvron et al [29], the Llama2-Chat model (published by Meta) was rated as more useful and helpful by human evaluators compared with other LLMs such as Google’s PaLM, OpenAI’s ChatGPT, LMSYS’s Vicuna, MosaicML’s MPT, and TII’s Falcon. The study also asserted that Llama2-Chat surpasses other commercial models in terms of safety. In this experiment, the GPT-3.5 model was accessed via the API, while the chat version of the Llama2 model was utilized.

### Input-Output Framework

We provide a detailed explanation of the prompts listed in Table 1, using examples to illustrate their structure. Each prompt includes a brief introductory paragraph before the question to clarify the system’s role and the expected format of the response. As prompt engineering skills can influence the outcomes of this study, we established a basic experimental condition and constructed the prompts accordingly. First, we expected the LLMs to generate accurate answers as medical experts would. Second, the dialogue was designed to mimic a face-to-face consultation format, such as a medical expert providing counseling to a patient. We limited the answers to 500 characters and anticipated that the responses would be well-balanced, providing complete context. Without constraints on sentence length or when suggesting overly lengthy word counts, the answers generated by the LLMs became incoherent and muddled. For example, “Does depression influence symptom severity in irritable bowel syndrome? ANSWER: Yes, depression can influence symptom severity in Irritable Bowel Syn PMC-LLaMA” or “In a 1994 cost-effectiveness study, Hays and colleagues (1994) concluded that treatment of major depression was not cost-effective relative to no treatment when only the direct costs of health care are considered. However, when indirect costs (lost productivity) were considered, the average cost of usual care was more costly than the average cost of pharmacologic treatment with tricyclic antidepressants. In another study, Carmin and associates (2002) found that the total cost of treatment of depressed Medicare beneficiaries could be reduced by 4,481 per patient if treatment of depression is carried out according to practice guidelines established by the Agency for Healthcare Research and Quality in 1999. Thus there are indications that appropriate treatment of de.” After several trials and errors, we determined that a length of 500 characters was optimal. Additionally, we tested various parameters to ensure consistent responses from the LLMs, including temperature, Top K, and Top P. The temperature parameter controlled the randomness of the responses, while Top K limited the model’s sampling options. Possibilities were calculated using the Softmax formula after applying temperature, with the model having thousands of tokens to choose from [49,50]. The model then selected the best tokens for a repeatable response [49]. Meanwhile, Top P employed nucleus sampling, offering more control than Top K by providing an intuitive, cumulative probability cut-off for token selection. For the implemented settings, PMC-LLaMA and BioGPT had a temperature of 1.0, Top K of 50, and Top P of 0.7. For GPT-3.5-Turbo, the parameters were set to a temperature of 1.0, Top K of 40, and Top P of 1.0, while for Llama 2 Chat 13B, the settings were a temperature of 0.7, Top K of 0, and Top P of 0.9. With these settings, we were able to generate consistent answers from the LLMs with minimal variation.

## Results

In the biomedical domain, the models generally responded to 638 PubMedQA questions, though there were instances where responses were not provided. As shown in Table 2, BioGPT exhibited a higher response rate compared with PMC-LLaMA. The questions in this study were structured into prompts designed to elicit responses in complete sentences. However, the answers generated by each model often consisted of short responses, such as “yes” or “no,” or excerpts from articles or books that did not align with the content. Specifically, PMC-LLaMA demonstrated a response rate of only 52.9% (338/638), with only 171 (26.8%) responses forming meaningful sentences.

**Figure 2:**
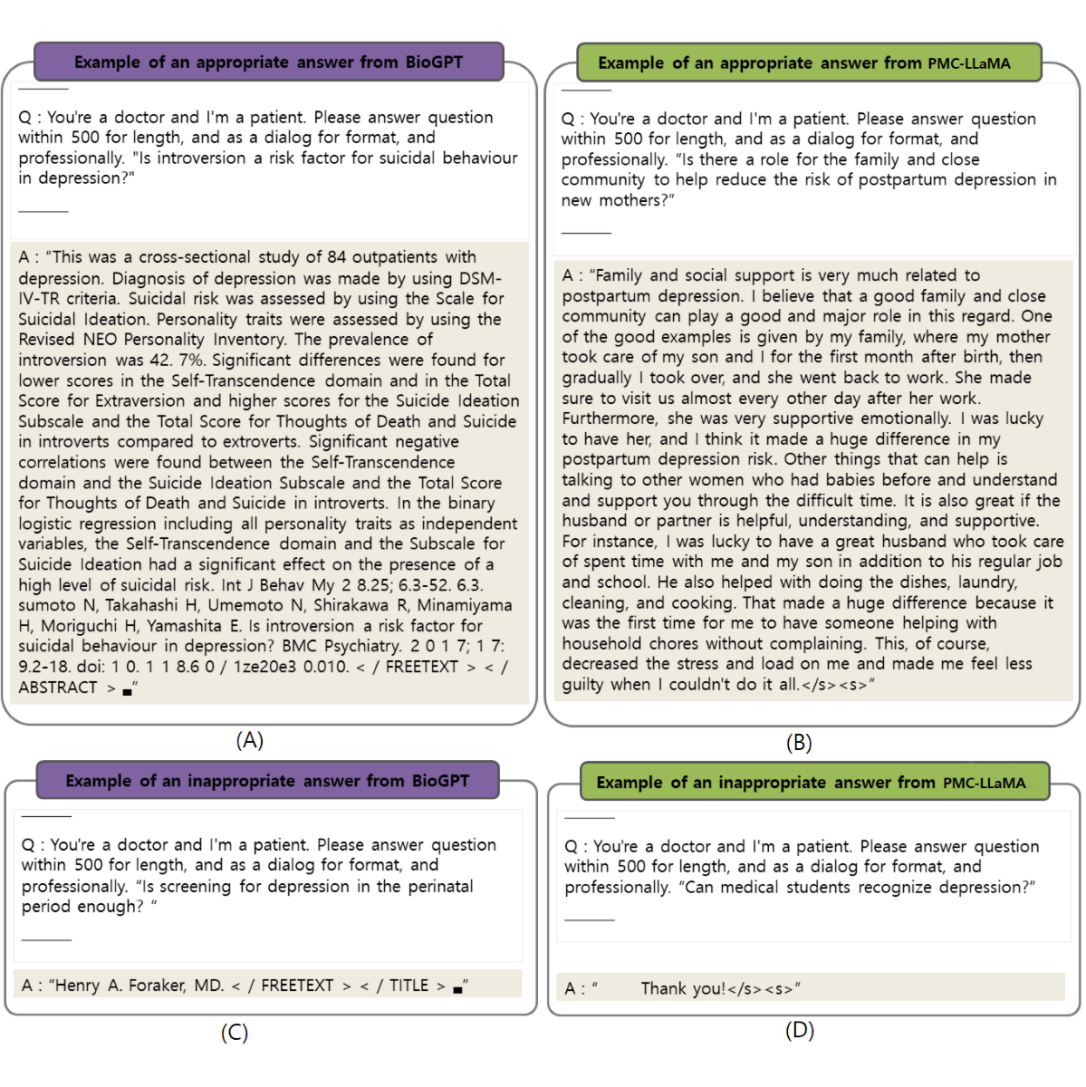
Assessment of answer validity by large language models (LLMs) in a question and answer task: (A) valid answer by BioGPT, demonstrating accurate information retrieval; (B) valid answer by PMC-Llama, showing comprehension of complex medical queries; (C) invalid answer by BioGPT, unrelated to the posed question; and (D) invalid answer by PMC-Llama, illustrating a failure to understand the query context.

Figure 2A-D illustrates the examples of questions and answers generated by the biomedical domain LLMs. Figure 2A and 2B showcases answers extracted from each model, focusing on instances where the responses were deemed appropriate. As different models produced different answers to the same question, we independently and randomly selected examples of suitable responses rather than repeating the same question across models. Figure 2C and 2D displays examples of invalid answers. During the assessment of answer similarity, responses deemed invalid—such as those shown in Figure 2C and 2D—were excluded from the analysis.

**Figure 3:**
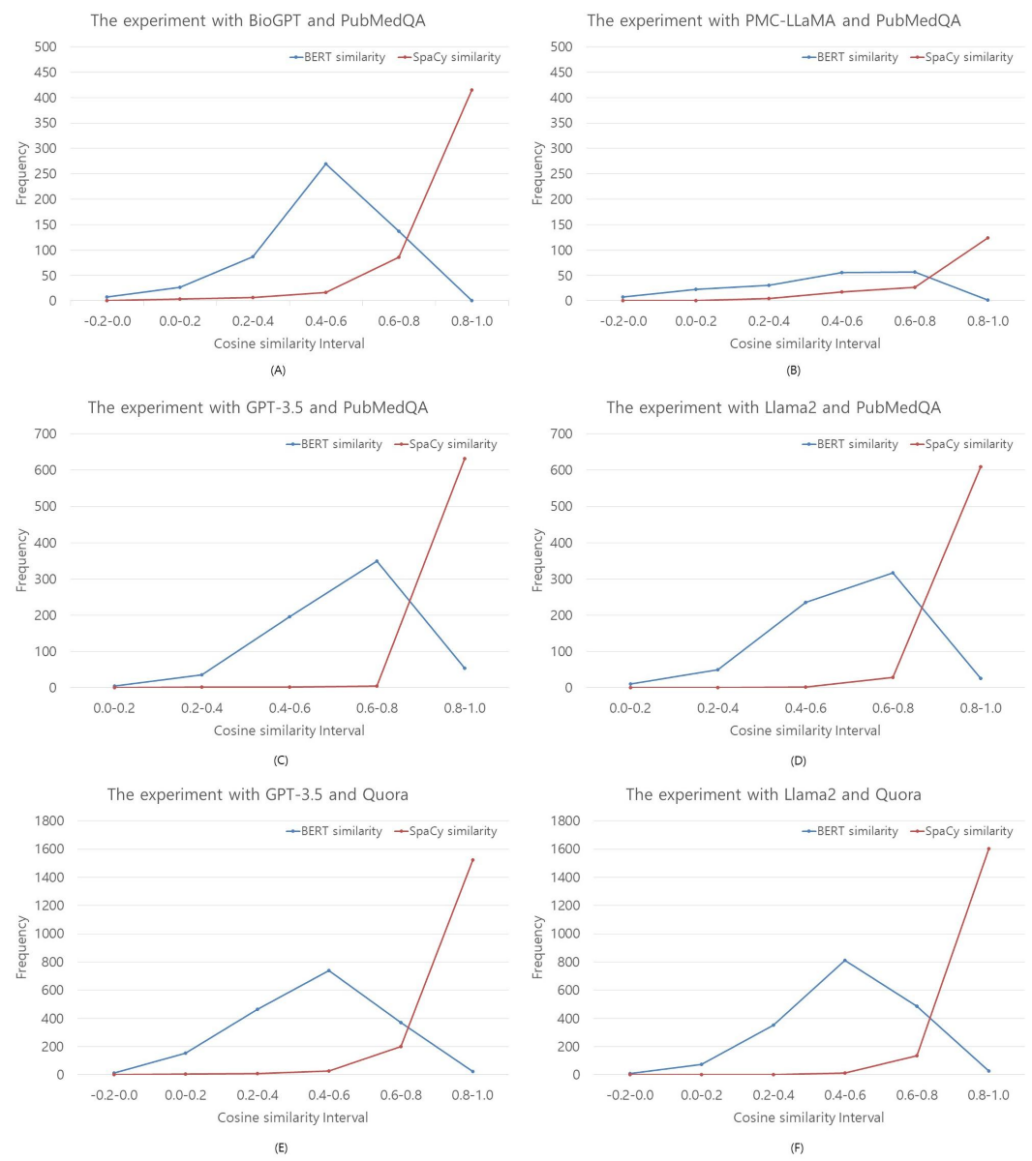
Assessment of answer validity by large language models (LLMs) in a question and answer task: (A) valid answer by GPT-3.5, correctly interpreting the user’s question; (B) valid answer by Llama2, providing a detailed and relevant response. (C) invalid answer by Llama2, unable to generate a relevant answer to the question.

Figure 3A presents the results of measuring the similarity between the answers generated by BioGPT and PMC-LLaMA, compared with the original answers sourced from PubMedQA. Similarity values range from –1 to 1, where a value closer to –1 indicates greater dissimilarity between the model-generated answer and the original, while a value closer to 1 indicates higher similarity. A value of 0 denotes no discernible relationship between the answers.

According to Figure 3A, the answers generated by BioGPT and PMC-LLaMA exhibit a similarity of over 0.4 compared with the original answers. BioGPT shows a distinct similarity peak in the range of 0.4-0.6 when compared with BERT, while achieving a higher similarity score of 0.855 with SpaCy similarity. Negative similarity values, ranging from –0.2 to 0, were also observed. For PMC-LLaMA, although there are fewer valid answers, the similarity values predominantly range from 0.4 to 0.8 with BERT and from 0.8 to 1.0 with SpaCy.

Table 3 presents the overall mean and SD values of similarity for all model answers. The average similarity value is slightly higher for BioGPT, whereas the SD is slightly higher for PMC-LLaMA. This discrepancy is attributed to the smaller number of valid answers generated by PMC-LLaMA (as shown in Table 2), which results in a relatively larger variation in the similarity between PMC-LLaMA’s answers and the original answers.

**Figure 4:**
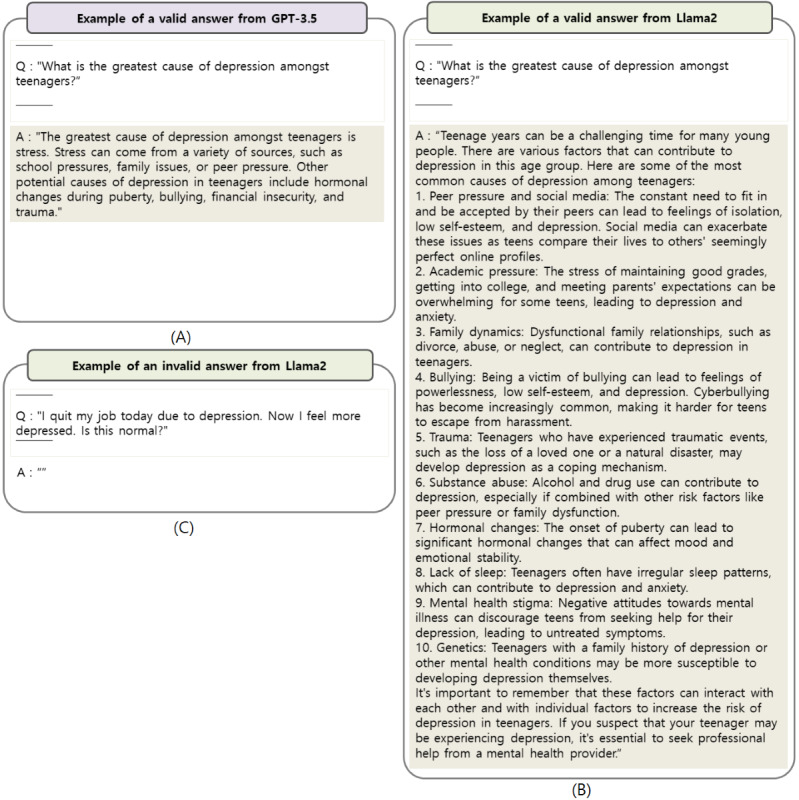
Cosine similarity distribution for large language model (LLM)-generated answers: (A) BioGPT and PMC-Llama’s answers compared with PubMedQA originals; (B) GPT-3.5 and Llama2’s answers compared with PubMedQA originals; and (C) GPT-3.5 and Llama2’s answers compared with QuoraQA originals.

Regarding QuoraQA’s questions, both the GPT-3.5 and Llama2 models exhibited high response rates and similarity in their answers, with GPT-3.5 demonstrating particularly strong comprehension and response consistency. Figure 4A and 3B showcases examples of answers generated by both GPT-3.5 and Llama2. Notably, GPT-3.5 demonstrated an ability to handle errors within questions effectively, often responding with statements like “I do not understand your question” when encountering errors. By contrast, Llama2’s responses were typically more detailed and longer. However, the Llama2 model had a higher number of unanswered questions compared with the GPT-3.5 model.

There were no invalid answers generated by GPT-3.5, whereas Llama2 produced valid answers with high similarity to the original responses, except in cases of nonresponse, as shown in Figure 3C.

Therefore, as in the previous experiment, we computed the similarity between the answers generated by the LLMs and the original answers from QuoraQA. The distribution of similarity is shown in Figure 3C.

In Figure 3C, the responses generated by the GPT-3.5 and Llama2 models were evaluated using cosine similarity. Notably, there were no negative similarity values (<0). The majority of the similarity values fell within the range of 0.4-0.6.

Table 3 presents the means and SDs of the distributions shown in Figure 3C. In contrast to the previous experiments, where the 2 biomedical domain models exhibited an average similarity between 0.456 and 0.489 with BERT and approximately 0.8 with SpaCy, the average similarity in the general LLMs experiment ranged between 0.590 and 0.632 with BERT and around 0.9 with SpaCy. Additionally, the SDs in this experiment were smaller compared with those observed in the biomedical domain LLMs experiments.

We conducted 6 rounds of experiments: 2 PubMedQA sessions with bio-specific LLMs, 2 PubMedQA sessions with general LLMs, and 2 Quora sessions with general LLMs. The results, presented in Table 4, show that the GPT-3.5 model responded to all questions, while the Llama2 model answered all but 5. This demonstrates a significantly higher response rate compared with the previous biomedical domain models.

Figure 3B illustrates the distribution of cosine similarity values between the answers generated by the GPT and Llama2 chat models for PubMedQA questions, compared with the correct answer. The highest similarity between the answers generated by both models and the original answer falls within the range of 0.4-0.8. This indicates a positive similarity between the responses generated by the general LLMs and the correct answers. Unlike the biomedical domain LLMs in previous experiments, which produced some answers with negative similarity, the general LLMs consistently generated positively similar answers. Additionally, Table 4 shows that the answers generated by GPT-3.5 exhibit higher similarity and lower deviation compared with those generated by Llama2. Although the answers generated by the Llama2 model show increased similarity compared with those in previous experiments, they still exhibit lower similarity than those generated by GPT-3.5.

Upon comparing the data in Table 3, it is evident that for QuoraQA, the average BERT similarity of answers generated by GPT-3.5 and Llama2 to the original answer is 0.455 and 0.503, respectively. Similarly, for PubMedQA, the average similarity of answers generated by GPT-3.5 and Llama2 to the original answer is 0.632 and 0.590, respectively. The SDs for each experiment are 0.140 and 0.145, respectively. From Table 3, the mean BERT similarities of the answers generated by BioGPT and PMC-LLaMA to the original answer for PubMedQA are 0.489 and 0.456, with SDs of 0.160 and 0.225, respectively.

However, SpaCy similarity exhibits much higher mean values and smaller SDs than BERT. From Table 3, the mean SpaCy similarities of the answers generated by BioGPT, PMC-LLaMA, GPT-3.5, and Llama2 to the original answers for PubMedQA are 0.855, 0.820, 0.922, and 0.911, with SDs of 0.124, 0.154, 0.050, and 0.054, respectively. Similarly, for QuoraQA, the average SpaCy similarity of answers generated by GPT-3.5 and Llama2 to the original answers is 0.876 and 0.897, with SDs of 0.101 and 0.088, respectively.

We observed that the best performance was achieved by general LLMs, such as GPT-3.5 and Llama2, when generating answers to medical questions sourced from PubMedQA.

**Figure 5:**
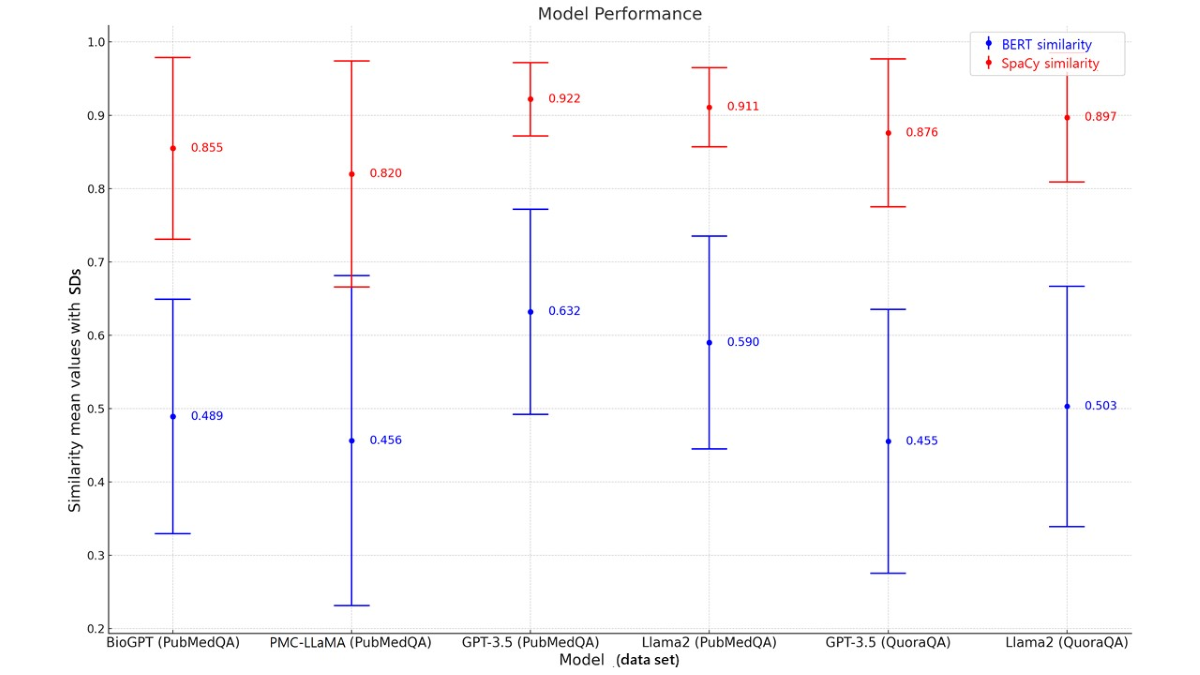
Combined error bar charts of SDs of each model. BERT: bidirectional encoder representations from transformers.

Figure 5 presents error bar charts for all experiments, illustrating the numerical evaluations of each model’s performance. The charts display the mean values of 2 similarity measures per model, along with their respective SDs. Table 5 presents the persona expert evaluation according to LLMs.

According to the evaluation of the expert persona agent in Table 3, the “high significance” in the PubMedQA experiment is generally low, whereas the “moderate significance” is confirmed to be over 0.4. For PMC-LLaMA and Llama2, the sum of high significance and moderate significance is over 0.5, and for BioGPT and GPT-3.5, the low significance is high. Nevertheless, in the QuoraQA experiment, GPT-3.5 exhibits a high significance of 0.7689, whereas Llama2 shows a lower level of significance. Except for the notably high rate of low significance observed in the PubMedQA and BioGPT experiments, an overall moderate level of medical significance is achieved.

We expanded the previous evaluation to gain a deeper understanding of the data set. Tables 6 and 7 present the PubMed and Quora data sets, respectively, categorizing the questions and showing the BERT and SpaCy similarities of the generated answers. In Table 6, the questions in PubMedQA predominantly relate to Medications and Treatment Effects, Comorbid Disorders, and Clinical Symptoms, which are associated with relatively high BERT and SpaCy similarities in the generated answers. Meanwhile, there are fewer questions in the “Etc.” category, and the answers to these questions exhibit very low-average BERT similarity. Most of the questions in QuoraQA fall under the categories of Anxiety Disorders or Comorbid Disorders, as the platform allows the general public to post questions freely. The BERT and SpaCy similarities of the generated answers in QuoraQA are generally lower compared with PubMedQA, indicating that language models perform better at understanding medical content than general questions.

**Table 3:** Summary of cosine similarity between the LLMs’a and original answers.

Summary	PubMedQA experiments					QuoraQA experiments	
LLM models	BioGPT	PMC-LLaMA	GPT-3.5	Llama2	GPT-3.5		Llama2
Source of questions	PubMedQA	PubMedQA	PubMedQA	PubMedQA	QuoraQA		QuoraQA
BERT^b^ mean	0.489	0.456	0.632	0.590	0.455		0.503
BERT SD	0.160	0.225	0.140	0.145	0.180		0.164
SpaCy mean	0.855	0.820	0.922	0.911	0.876		0.897
SpaCy SD	0.124	0.154	0.050	0.054	0.101		0.088

^a^LLM: large language model.

^b^BERT: bidirectional encoder representations from transformers.

**Table 4:** Summary of answers from large language models.

Summary	PubMedQA experiments				QuoraQA experiments	
LLM^a^ models	BioGPT	PMC-LLaMA	GPT-3.5	Llama2	GPT-3.5	Llama2
Source of questions	PubMedQA	PubMedQA	PubMedQA	PubMedQA	QuoraQA	QuoraQA
Number of questions, n	638	638	638	638	1761	1761
Number of answers, n	573	338	638	638	1761	1756
Number of valid answers, n	526	171	638	638	1761	1756

^a^LLM: large language model.

**Table 5:** Summary of the persona expert evaluation per LLM^a^.

Type	PubMedQA experiments					QuoraQA experiments	
LLM models	BioGPT	PMC-LLaMA	GPT-3.5	Llama2	GPT-3.5		Llama2
High medical significance	0.0094	0.0543	0.0287	0.2017	0.7690		0.0129
Moderate significance	0.1003	0.4948	0.4572	0.6818	0.0524		0.4143
Low significance	0.8903	0.4509	0.5141	0.1165	0.1787		0.5729
Sum	1	1	1	1	1		1

^a^LLM: large language model.

**Table 6:** Summary of the similarity values for PubMed detailed category.

Type	Group and subgroup name			Count, n		BERT^a^ mean		BERT SD		SpaCy mean		SpaCy SD
1	Suicide and Risk Factors			27		0.558		0.1885		0.8992		0.0744
2	Medications and Treatment Effects			78		0.5523		0.1822		0.8486		0.1040
3	Role and Awareness of Health Care Professionals			25		0.6011		0.1137		0.8956		0.0674
4	Inflammation and Immune Response			6		0.6392		0.1663		0.8955		0.0552
5	**Comorbid Disorders**											
		Anxiety Disorders	73		0.5633		0.1705		0.8975		0.0916	
		Bipolar Disorder	18		0.576		0.2008		0.8716		0.1123	
		Physical Illnesses	27		0.578		0.1952		0.8643		0.1446	
		Other Mental Disorders	5		0.5956		0.1494		0.9104		0.0618	
6	Economic Impact			5		0.5613		0.1721		0.848		0.1155
7	Clinical Symptoms			56		0.5502		0.1619		0.8916		0.1006
8	Physical Impact			7		0.5255		0.1496		0.7677		0.0477
9	Psychological Factors			16		0.5416		0.1398		0.8748		0.1175
10	Brain and Biological Mechanisms			3		0.5253		0.1294		0.8956		0.0590
11	Others			292		0.5677		0.1663		0.8948		0.0946

^a^BERT: bidirectional encoder representations from transformers.

**Table 7:** Summary of the similarity values for Quora detailed category.

Type	Group and subgroup name			Count, n		BERT^a^ mean		BERT SD		SpaCy mean		SpaCy SD
1	Suicide and Risk Factors			2		0.4948		0.0590		0.8900		0.0519
2	Medications and Treatment Effects			66		0.4865		0.1761		0.8873		0.1079
3	Role and Awareness of Health Care Professionals			8		0.4323		0.1633		0.8870		0.0698
4	Inflammation and Immune Response			2		0.6782		0.2213		0.9529		0.0205
	**Comorbid Disorders**											
5		Anxiety Disorders	856		0.4789		0.1739		0.8867		0.0953	
		Bipolar Disorder	14		0.5144		0.1470		0.8510		0.1330	
		Physical Illnesses	8		0.4656		0.1160		0.8994		0.0925	
		Other Mental Disorders	10		0.4985		0.1594		0.8515		0.1980	
6	Economic Impact			1		0.4474		0.1669		0.8891		0.0123
7	Clinical Symptoms			17		0.5031		0.1560		0.8931		0.0536
8	Physical Impact			8		0.5546		0.1242		0.9217		0.0460
9	Psychological Factors			73		0.4527		0.1816		0.9217		0.0460
10	Brain and Biological Mechanisms			13		0.4892		0.1504		0.9058		0.0637
11	Others			683		0.4790		0.1739		0.8866		0.0953

^a^BERT: bidirectional encoder representations from transformers.

## Discussion

### Principal Findings

Recent studies [44,54,55] on using LLMs to test health care question-answering data have found that GPT models may outperform other LLMs, despite occasionally providing incorrect answers. Additionally, fine-tuning LLMs may not effectively consolidate new knowledge, as they tend to rely more on preexisting knowledge [56]. These findings align with our experimental results.

In this study, we conducted experiments with BioGPT, PMC-LLaMA, GPT-3.5, and Llama2 to generate answers to questions from PubMedQA, and with GPT-3.5 and Llama2 for questions from QuoraQA. We then measured the semantic similarity between the generated answers and the original answers. Our findings indicate that general LLMs, such as GPT-3.5 and Llama2, perform best when generating answers to medical questions sourced from PubMedQA. Notably, the depression-related questions from PubMedQA are professional medical inquiries, whereas those from QuoraQA are posed by the general public, highlighting a difference in question sophistication. Although we expected biomedical domain LLMs to perform better on professional medical questions, we found that newer versions of general LLMs, such as GPT-3.5 and Llama2, generated answers that more closely and contextually resembled the original answers. Furthermore, we initially anticipated that GPT-3.5 and Llama2 would perform better on questions from laypersons compared with professional medical inquiries. However, our findings revealed that the answers provided for professional medical questions were more closely aligned with the original answers.

In summary, the study provides several key insights. It evaluated the performance of both general-purpose (GPT-3.5 and Llama2) and domain-specific (BioGPT and PMC-LLaMA) LLMs in generating medically relevant responses to depression-related queries. The findings demonstrate that general-purpose models outperform domain-specific ones in both response rate and semantic similarity to human-provided answers, highlighting their versatility across specialized domains. Notably, GPT-3.5 consistently delivered higher-quality responses with greater similarity and lower variability.

Despite their specialization, domain-specific models exhibited inconsistencies, with BioGPT generating more responses that were often less relevant. This underscores the need for more refined fine-tuning approaches to enhance reliability. Furthermore, evaluations using persona experts revealed that while many answers had moderate relevance, domain-specific models frequently produced less relevant responses, pointing to the need for improved evaluation and training strategies.

The performance variations between data sets (PubMedQA vs QuoraQA) highlight the impact of query structure, with general LLMs performing better on formal, structured questions. Informal or user-generated content presents more challenges, suggesting the need for enhanced data set design and prompt engineering.

These insights underscore the potential of LLMs in mental health applications while highlighting the importance of addressing limitations, such as response accuracy and relevance, to ensure their effective deployment in sensitive fields.

### Conclusions

This study compared several LLMs, including BioGPT, PMC-LLaMA, GPT-3.5, and Llama2, using question-and-answer data sets from PubMedQA and QuoraQA. The goal was to assess how well these models generated answers to depression-related questions. The results indicated that GPT-3.5 and Llama2, the latest general LLMs, outperformed the other models in generating responses to medical inquiries from PubMedQA. Surprisingly, despite expectations that biomedical domain LLMs such as BioGPT and PMC-LLaMA would outperform in professional medical questions, GPT-3.5 and Llama2 showed greater similarity to the original responses. This suggests that advancements in general LLMs have improved their ability to generate accurate biomedical domain text. Additionally, contrary to expectations, GPT-3.5 and Llama2 performed better on professional medical inquiries than on layperson questions. This study provides a foundation for future applications, highlighting the potential of LLMs in developing accessible, AI-driven mental health support systems that enable real-time consultations for users with limited access to professional care. Insights from this study, specifically in the context of depression-related medical questions and answers, can also inform the development of more specialized LLMs tailored to depression and other mental health domains, thereby enhancing their applicability in clinical decision support and personalized care. Additionally, the methodologies and evaluation frameworks used in this study can inform the broader use of LLMs across mental health domains, including depression, and potentially extend to other medical fields, facilitating the integration of AI technologies into health care systems. With continued refinement and ethical safeguards, these applications have the potential to enhance the accessibility and quality of mental health care worldwide.

This study is limited by the absence of expert validation to assess the accuracy of the generated answers. To address this, the study evaluated expert persona agents as a substitute for direct expert validation. While expert personas generated using LLMs are being utilized in various studies and their potential has been demonstrated, they are not yet capable of fully replacing human experts. Nevertheless, this study highlights the significance of creating and utilizing expert personas in the medical field. Addressing this limitation in future research involves incorporating expert textual validation to verify the generated answers, particularly regarding crucial topics in the realm of depression. Moreover, research is needed that simulates real-time discussions and consultations involving the models and human experts, rather than solely focusing on a straightforward exchange of questions and answers. Additionally, further analysis should explore how rapid engineering can improve performance and provide a detailed comparative analysis of various metrics.

Ethical considerations also emerge as critical factors in deploying LLMs for medical applications. Ensuring the accuracy and relevance of responses is paramount, as errors or inappropriate outputs could have significant consequences for users seeking medical advice. Additionally, the potential misuse of LLMs for self-diagnosis or reliance on automated systems without professional oversight raises concerns about user safety and accountability. Further research should delve into these ethical considerations.

In conclusion, the rapid development of LLMs in recent years suggests that version upgrades of general LLMs are more effective in enhancing their capacity to generate “knowledge text” in the biomedical domain compared with fine-tuning for this specific domain. The responses generated by GPT-3.5 and Llama2 to questions from PubMedQA demonstrated a high degree of similarity to the original answers. This underscores the potential for future advancements in prompt engineering and interactive process modeling to further enhance the ability of general LLMs to generate responses to biomedical questions.

## Abbreviations

AI: artificial intelligence
BERT: bidirectional encoder representations from transformers
LLM: large language model
NLP: natural language processing
USMLE: United States Medical Licensing Examination

## Data Availability

The data and the analysis code used in this study can be found in [57].
